# An Electrifying Case of a Broken Heart Syndrome

**DOI:** 10.7759/cureus.29476

**Published:** 2022-09-22

**Authors:** Ana Rita Moura, Bruno Castilho, Kevin Domingues, Vitor Martins

**Affiliations:** 1 Cardiology, Hospital Distrital de Santarém, Santarém, PRT

**Keywords:** biventricular takotsubo cardiomyopathy, permanent pacemaker implantation (ppm), heart failure, pacemaker lead displacement, takotsubo cardiomyopathy (ttc)

## Abstract

Takotsubo syndrome (TTS) describes an acute and transient left ventricular (LV) dysfunction that, although not obligatory, is many times associated with an underlying emotional, physical, or combined trigger. We describe a rare case of an 80-year-old female who developed TTS after pacemaker implantation in the context of a complete atrioventricular block (CAVB). During the patient's workup, right ventricular (RV) lead dislodgment was found. She developed acute heart failure symptoms 12 hours after device implantation with transthoracic echocardiogram showing de novo severe systolic biventricular dysfunction with dyskinesia of the apical segments and hyperdynamic contractility of the basal segments of both ventricles. Coronarography was normal, and left ventriculography demonstrated apical ballooning. TTS was then considered the most probable diagnosis. The patient received supportive care with diuretics, beta-blocker, and angiotensin-converting enzyme inhibitor (ACEI); an RV lead repositioning was also done. After four weeks, left ventricular function had fully recovered, confirming the diagnosis. This is a rare case of a post-pacemaker implantation TTS with concomitant lead dislodgment that can be assumed as a likely contributing factor. This report emphasizes that, although rare, TTS should be considered in the differential diagnosis of patients with acute heart failure development after pacemaker implantation.

## Introduction

Takotsubo syndrome (TTS) describes an acute and transient left ventricular (LV) dysfunction that, although not obligatory, is many times associated with an underlying emotional, physical, or combined trigger. While the exact mechanism of TTS remains unknown, it is hypothesized that it can result from an excess of circulatory catecholamines secondary to an exaggerated sympathetic response to a stressor [1]. Patients typically present with symptoms and signs mimicking those of an acute myocardial infarction and/or heart failure [1]. In its most common form, the heart assumes a transient typical aspect during systole characterized by left ventricle (LV) apical ballooning due to akinesis or hypokinesis of the mid-to-distal segments and hyperkinesis of its basal segments [2]. Isolated LV involvement is the most common variant; however, right ventricular (RV) involvement is gaining increased recognition (25%-42%) [3].

Complications related to pacemaker implantation are relatively common, occurring in a total of 5.3% of the patients in the first six months post-procedure [4]. On the other hand, although already described in a few cases, TTS following pacemaker implantation is a very uncommon [5] but potentially serious intercurrence.

## Case presentation

**Figure 1:**
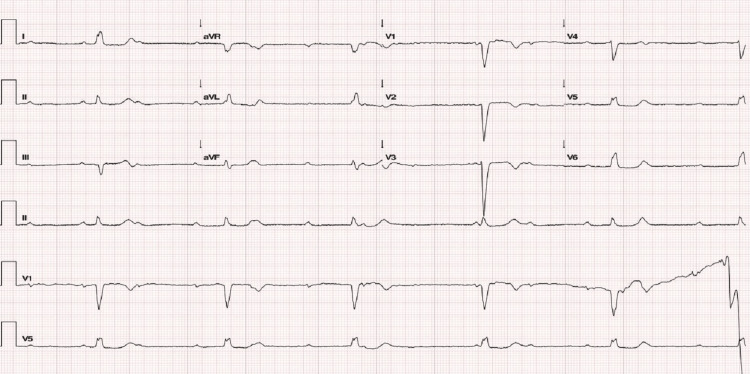
Electrocardiogram revealing complete atrioventricular block with ventricular escape rhythm of 33 bpm. aVF: arteriovenous fistula; aVL: augmented vector left; aVR: aortic valve replacement; bpm: beats per minute.

An 80-year-old female, with known hypertension and dyslipidemia treated with amlodipine 5 mg once daily (od) and rosuvastatin 10 mg od, presented to the emergency department complaining of extreme fatigue that started a week before. There was no history of syncope, and she wasn't on any anti-arrhythmic drugs. On examination, the patient was hemodynamically stable, bradycardic, and eupneic; physical examination was unremarkable. Electrocardiogram (ECG) revealed complete atrioventricular block (CAVB) with ventricular escape rhythm at 33 beats per minute (bpm) (Figure 1).

Her blood test results had no relevant changes besides a high B-type natriuretic peptide (BNP) (1188 pg/mL), including no hydroelectrolytic abnormalities. Chest X-ray had no significant alterations. Transthoracic echocardiography (TTE) revealed no dilation of cardiac chambers, good biventricular systolic function, and no changes in segmental contractility of the LV (Video 1).

**Video 1:** Transthoracic echocardiography at patient's admission showing no dilation of cardiac chambers, good biventricular systolic function, and no left ventricular segmental contractility abnormalities.

The patient was hospitalized with a diagnosis of CAVB. After the exclusion of reversible causes, a dual-chamber pacemaker was implanted the next day without complications. A chest X-ray at the end of the procedure revealed stable pacemaker lead positions with the ventricular electrocatheter in the RV apex. Twelve hours later, the patient complained of new-onset dyspnea. There was no history of chest pain. Pulmonary auscultation revealed bi-basal rales. Post-implantation ECG showed sinus rhythm and a ventricular paced rhythm at 80 bpm, with a corrected QT (QTc) of 416 ms. A TTE was performed showing diffuse hypokinesia of the mid and distal segments of the LV and the RV free wall, as well as evident hyperkinesia of the basal segments of both ventricles. LV systolic function was severely reduced with an LV ejection fraction of 20% (Video 2).

**Video 2:** Transthoracic echocardiography performed after new-onset heart failure symptoms, showing hypokinesia of all mid and apical segments of both ventricles, with severely reduced left ventricular ejection fraction.

**Figure 2:**
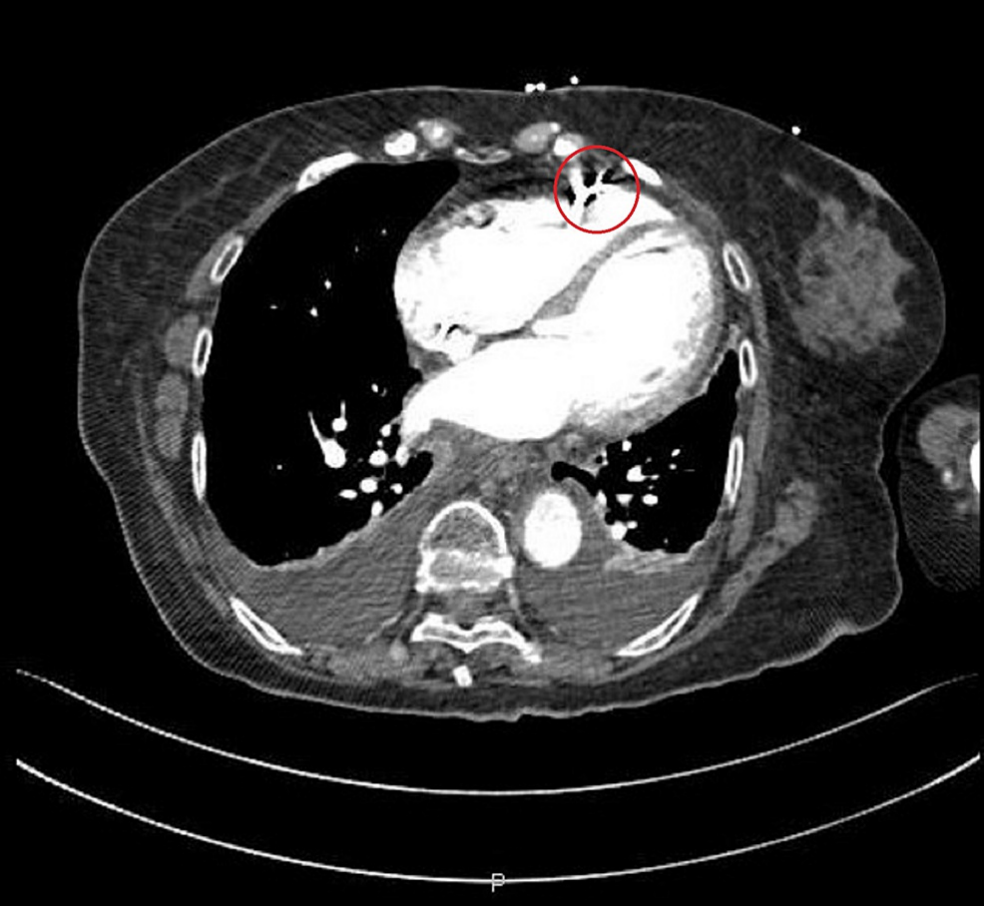
Thoracic CT scan revealing dislodgment of the right ventricular lead and bilateral pleural effusion.

Blood workup revealed an elevated high-sensitivity troponin of 3463 ng/mL (for a normal of <15.6 ng/mL), BNP of 3550 pg/mL, and D-dimers of 2800 ng/mL. Given the elevation of the latter and the new RV dysfunction, a CT scan was performed, which showed no signs of pulmonary embolism but, instead, revealed dislodgment of the ventricular electrocatheter that was inserted at the RV free wall with no extravasation of contrast to the pericardial space (Figure 2).

**Figure 3:**
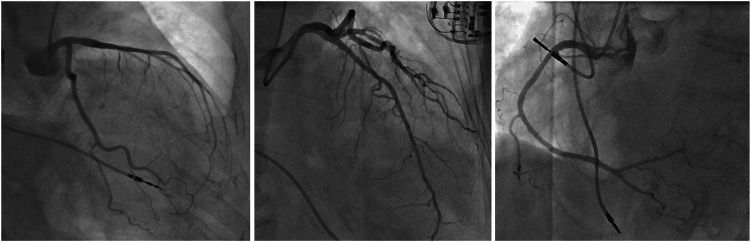
Coronary angiography showing no coronary stenosis.

A pacemaker check was performed, revealing elevation of both threshold and sensing ventricular values; there were no records of capture failure from the ventricular lead, with the patient having 100% of RV pacing since device implantation. Lead repositioning was then executed. Coronary angiography was performed on the next day showing no coronary stenosis (Figure 3), and left ventriculography revealed an apical ballooning pattern (Video 3).

**Video 3:** Left ventriculography with apical ballooning pattern.

TTS was assumed as the most probable diagnosis. Accordingly, the patient was treated with beta-blocker, angiotensin-converting enzyme inhibitor, and diuretics. She was discharged on bisoprolol 2.5 mg once daily and ramipril 10 mg once daily. A follow-up TTE at one month after discharge revealed an LV ejection fraction of 54% and resolution of regional wall motion abnormalities (Video 4); in the absence of other identifiable causes, this reassured TTS diagnosis.

**Video 4:** Re-evaluation transthoracic echocardiogram showing recovery of the left ventricular systolic function and resolution of regional wall motion abnormalities.

## Discussion

Available reports in the literature regarding post-pacemaker TTS between 2006 and 2022 (Table 1) show that the occurrence of this entity is more common in female patients [5-14], mainly in the context of CAVB [5-7,10,13,15], with symptom onset delay after device implantation between 10 minutes and seven days [7,13], and only a few identify procedure-related anxiety as the most likely underlying emotional trigger [6,14]. Other authors refer to the use of isoprenaline (catecholamine) [10,16] and the primary conduction disturbances [17] as potential contributors to TTS development.

**Table 1:** Cases of Takotsubo syndrome after pacemaker implantation, review of the literature (2006-2022). AVB: atrioventricular block; LVEF: left ventricle ejection fraction; PM: pacemaker; TTS: Takotsubo syndrome.

Age (years)	80	89	77	77	83	78	65	77	67	64	61	76	86	84	72
Gender	Female	Female	Female	Female	Female	Female	Female	Male	Female	Female	Female	Female	Male	Female	Female
Previous heart disease	No	No	No	No	No	No	No	No	No	No	No	No	No	No	No
Rhythm disturbance	Complete AVB	Complete AVB	Complete AVB	Mobitz I second-degree AVB	Tachy-brady syndrome	Complete AVB	Complete AVB	Complete AVB	High-grade AVB	Sinus node dysfunction	Complete AVB	Sinus node dysfunction	Complete AVB	Complete AVB	2:1 AVB
Use of isoprenaline/catecholamine	No	No	No	No	No	No	Yes	No	No	No	No	No	Yes	No	No
Type of PM	Dual chamber	Dual chamber	Dual chamber	-	Dual chamber	Dual chamber	Dual chamber	Dual chamber	Dual chamber	Dual chamber	Dual chamber	Dual chamber	-	Dual chamber	Dual chamber
Procedure-related anxiety	No	No	No	No	No	Yes	No	No	No	No	No	No	No	No	Yes
Hours after PM implantation until TTS diagnosis	12	0.17	72	12	-	4	Few	Few	24	24	24	24	8	168	-
LVEF at diagnosis, %	56	62	75	>60	55	-	50	76	Normal	-	Normal	-	-	Normal	Normal
LVEF after TTS diagnosis, %	20	38	27	20	40	13	25	32	Mildly to moderately reduced	40	Severely depressed	24	-	30	40
PM implantation-related complication	Lead dislodgment	No	No	No	No	No	Pneumothorax	No	No	No	No	No	No	No	No
Time of follow-up, weeks	4	8	12	5	8	3	1	8	14	14	3	-	4	20	16
LVEF recovery	Yes	No	No	Yes	Yes	Yes	Yes	Yes	Yes	Yes	Yes	-	Yes	Yes	Yes
Death	No	No	No	No	No	No	No	No	No	No	No	No	No	No	No
Year of publication	2022	2006	2006	2007	2009	2010	2011	2011	2011	2011	2014	2016	2016	2018	2018
Reference	Current case	7	7	8	9	6	10	15	11	11	5	12	16	13	14

In our case, given the hemodynamic stability of our patient, there was no need for the use of catecholamines before device implantation; additionally, there were no echocardiographic significant alterations at admission, and the patient evolved with heart failure symptoms only several hours after device implantation. Although it's not possible to exclude its contribution, this makes CAVB per se a less likely cause to the development of TTS. Consequently, the ventricular lead dislodgment seems to prevail as a relevant underlying stressor to take into consideration, which makes it a unique case. Different underlying pathophysiological mechanisms can be hypothesized such as myocardial inflammation due to partial perforation of the RV free wall or the marked LV dyssynchrony induced by improper positioning of the RV lead.

## Conclusions

In conclusion, our case report emphasizes that TTS, although rare, should be considered a potential complication of pacemaker implantation in patients evolving with symptoms of acute heart failure. A correct diagnosis is important to promptly identify potential associated complications such as LV outflow obstruction and severe arrythmias and proceed with adequate treatment. The rarity of this entity in this context and the possibility of its typical ECG changes being masked by ventricular pacing rhythm may preclude an early recognition making the diagnosis difficult and dependent on a high clinical suspicion. In these cases, lead dislodgment can be an underlying trigger and should be considered.
